# Stability investigation of air-dried olive ribo nucleic acids for metavirome studies

**DOI:** 10.1186/s13007-022-00846-6

**Published:** 2022-02-20

**Authors:** Leila Mirzaei, Abbas Yadollahi, Maryam Jafarkhani Kermani, Masoud Naderpour, Ali Asghar Zeinanloo

**Affiliations:** 1 Department of Horticultural Sciences, Faculty of Agriculture, Tarbiat Modares University, P. O. Box: 14115-111, Tehran, Iran; 2 Department of Tissue and Cell Culture, Agricultural Biotechnology Research Institute of Iran (ABRII), Agricultural Research, Education and Extension Organization (AREEO), P. O. Box: 31359-33151, Karaj, Iran; 3 Seed and Plant Certification and Registration Research Institute (SPCRI), Agricultural Research, Education and Extension Organization (AREEO), P. O. Box: 31535-1516, Karaj, Iran; 4 Temperate Fruit Research Center, Horticultural Research Institute, Agricultural Research, Education and Extension Organization (AREEO), P. O. Box: 31585-4119, Karaj, Iran

**Keywords:** Olive, RNA integrity, RNA quality, Metavirome studies

## Abstract

**Background:**

The application of ribo nucleic acids for molecular studies requires high integrity and quality of extracted total RNA samples. In addition, the need to transfer RNA samples at room temperature without special treatments such as ice and liquid nitrogen storage according to international transport laws highlights the importance of low cost alternative methods such as RNA air-drying, lyophilisation and transportable agents. In this study, the quality and quantity of air-dried RNA samples from leaf, petiole and bark tissues of different olive genotypes using several RNA extraction methods were compared with lyophilized ground leaves and RNAlater-stored tissue samples before precipitation. The quality of RNA and prepared libraries were checked by several techniques including agarose and polyacrylamide gel electrophoresis, Agilent quality control, RT-PCR amplification of housekeeping and viral genes and high throughput sequencing.

**Results:**

Although RNA value varied amongst cultivars, RNA extraction with TRIzol™ Reagent in fresh extractions and samples stored in RNAlater before RNA extraction resulted in 455.26 ng/µL and 63.46 ng/µL (mean value of cultivars) as the highest RNA concentration averages, respectively. RNA samples extracted by TRIzol™ Reagents and stored for a short term at – 80 °C before air-drying showed the third highest concentration (44.87 ng/µL). The synthesized cDNAs quality for PCR amplification of housekeeping genes (*Rbc 1* and *Nad 5*) and partial genomes of *Arabis mosaic virus* and *Cucumber mosaic virus* showed satisfactory results in RNA samples extracted by TRIzol™ Reagents despite its variation amongst cultivars.

**Conclusions:**

Considering the difficulties in the extraction of high quality and quantity RNA in olive for molecular analyses, this study demonstrated that RNA extraction method based on TRIzol™ Reagent can be considered for virobiome studies of both fresh and air-dried samples.

## Background

In comparison with DNA, the physico-chemical stability is more prominent in the case of RNA. Typically, RNA is rapidly degraded and it is vulnerable to hydrolysis by ubiquitous ribonucleases and/or divalent cations. Ribonucleic acids are degraded in several hours or days at room temperature even in the absence of RNase [1]. RNA detection methods require fresh tissues with minimum processing time and storing at ultralow temperatures usually at − 20 °C or even − 80 °C [2] and still their RNAs stability over time is not guaranteed [1].

Long-distance shipment of biological samples for international exchange between collaborators or analysis is inevitable [2]. According to the International Air Transport Association (IATA), [Acceptance checklist for dry ice. (Accessed January 1, 2015). Available at: http://www.iata.org/whatwedo/cargo/dgr/Documents/Acceptance-Checklist-Dry-Ice2014-EN.pdf], there are several limitations for routine transfer of biological materials using dry ice where temperature may still compromise the integrity of the RNA being shipped. RNA molecules are inherently sensitive to a number of factors such as heat, oxidation, pH alterations, and especially cellular RNases [3]. Lipid modification is present at the terminal ends of a linear RNA sequence for covalently linkers [1] and mostly RNA integrity declines in tissues which are notably higher in lipid content [4]. Since olive is highly rich in oil, RNA quality could be a concern. Seyednejad et al. [5] stated that fatty acids in olive leaves especially in native cultivars are higher, though it is around 10% but increases during olive fruit ripening. Lipid content around 35% of dry weight is really interfering [4].

Regarding viral diseases of plants, RNA viruses are still the most prevalent disease-causing agents particularly in deciduous plants [6]. Virus detection and identification in perennial plants are influenced by several factors [7]. Indeed, the specific and robust methods based on viral genomes are crucial for early detection of plant viruses [6]. Tremendous efforts have been put into RNA sequencing for a vast number of pathological samples [3]. Genome of most plant viruses are single- or double-stranded RNA with sense and/or antisense strands [1].

In the lyophilisation technique, RNA solvent, which is diethyl pyrocarbonate (DEPC) treated deionized water, is typically removed from frozen sample via sublimation [1]. Lyophilisation possesses two types of stresses namely, freezing and drying both of which are known to damage nucleic acids such as non-viral vectors or plasmid DNAs. [1]. It is an applicable tool for tissue samples rich in polyphenols and polysaccharides because it diminishes the RNA contamination mediated by nuclease and protease activities [2]. Moreover, RNA stabilization of tissues at non-cryogenic temperatures applying cell-penetrable fixatives such as RNAlater for short-term storage has recently been used as an alternative technique [8]. Air-drying of RNA in a suspension of yeast at 50 °C resulted in a nucleic acid (about 5% of that initially present) characterized by a relatively high ratio of adenine/guanine [9]. The mRNA is also conserved on rehydration after complete drying of drought-tolerant moss *Tortula ruralis* (Hedw.) [10].

Several limitations such as low antigen titre specifically in phloem restricted viruses [11, 12], irregular distribution of viruses in plant tissues [13], high temperature in sampling season [14], antiviral secondary metabolites like iridoid glycosides compounds [15], and cellular components such as oil, polysaccharide and polyphenols influence the accuracy of viral detection in olive [16, 17]. Therefore, the extraction and maintenance of high quality and integrity RNA is challenging [17] and it is basically required for reverse transcription-polymerase chain reaction (RT-PCR) amplification of viral targets [18]. In woody plants rich in secondary metabolites e.g., glycosides, polyphenols, and polysaccharides, which normally co-purify with nucleic acids, enzymatic reactions in dsRNA extraction are also inhibited [16, 19–21].

In the present study, some total RNA extraction methods including two modified TRIzol- and CTAB-based methods and two commercial RNA extraction kits (i.e., Ribospin Plant RNA Extraction and Spectrum Plant Total RNA Kits) were used for RNA extraction from different olive genotypes. The results were then compared to RNAs derived from lyophilized tissues and RNAlater immersion samples which later precipitated with Spectrum plant total RNA kit to obtain the highest quality and integrity RNA.

## Results

### The effect of plant tissue on RNA integrity and quality

The results of RNA extraction and contamination in two different tissue types are shown in Table 1. These indicate that extraction from leaf tissue generates RNA with higher concentration and less contamination (Table 1).

**Table 1:** Comparison of integrity and purity of RNAs extracted from different tissues of olive cultivar ‘Conservolia’ (Mean  ±  SE)

Tissue type	RNA concentration (ng/µL)	A260/280	A260/230
Stem cambium	33.520 ± 0.60	1.935 ± 0.06	0.12 ± 0.04
Leaf	525.68 ± 0.93	2.169 ± 0.05	1.202 ± 0.09

### Gel electrophoresis of RNA and PCR products

**Fig. 1:**
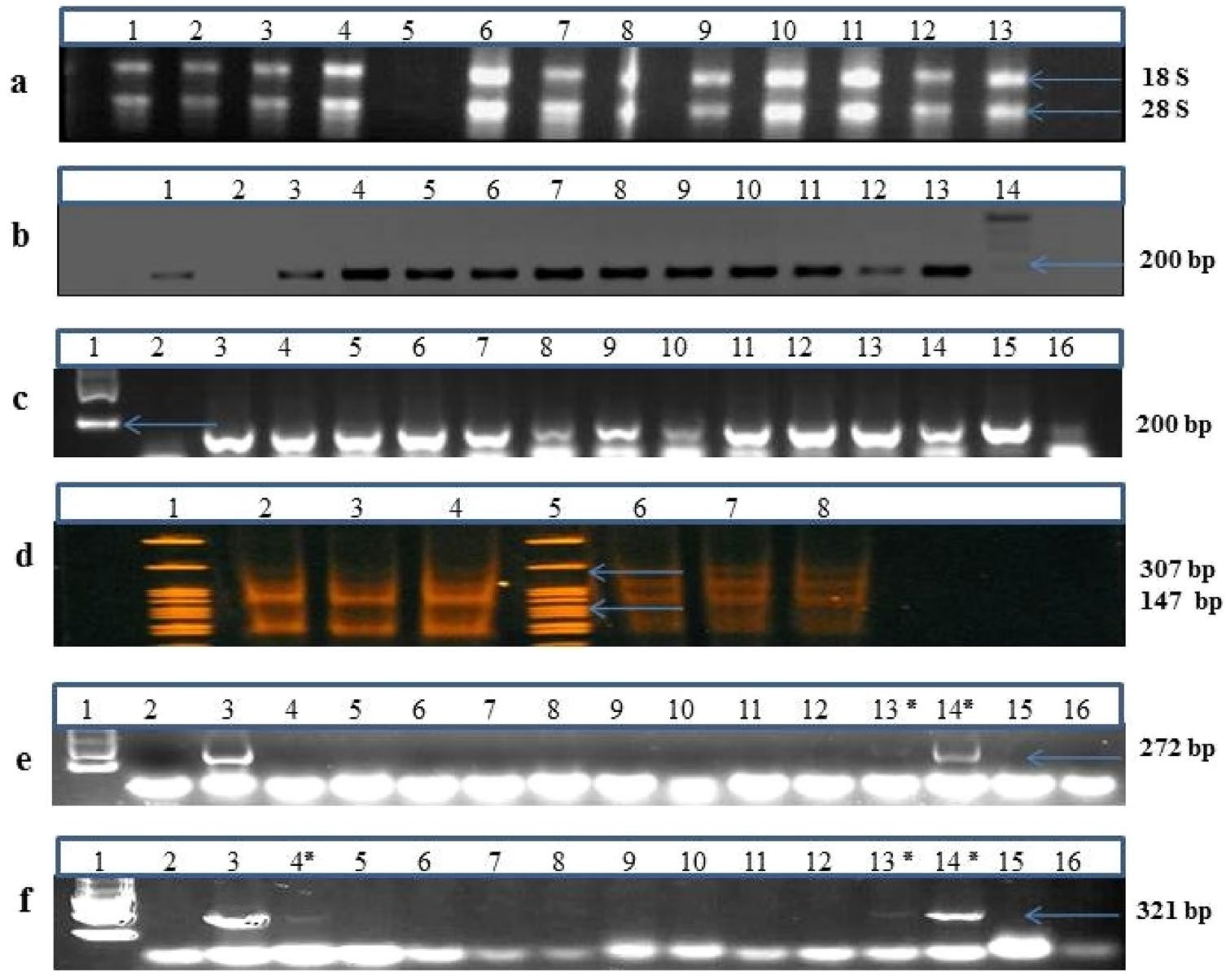
Assessment of RNA quality by electrophoresis, the synthesized cDNAs quality for amplification of housekeeping genes (*Rbc 1* and *Nad 5*), the prepared libraries, and partial amplification of viral genomes by RT-PCR. **a** Electrophoresis of RNAs on 1.5% agarose gel extracted from fresh tissues of olive cultivars ‘Dire’, ‘Meshkat’, ‘Conservolia’, ‘Arbequina’. **b**, **c** Partial amplification of 184 bp and 181 bp genomic fragments of *Rbc 1* and *Nad 5* genes, respectively using the cDNAs that synthesized from TRIzol extracted RNAs of cultivars ‘Dire’, ‘Meshkat’, ‘Conservolia’ and ‘Arbequina’. **d** Electrophoresis pattern of libraries prepared according to NEBNext^®^ Ultra™ II multiplex small RNA Library Prep set (NEW ENGLAND BioLabs, USA) for cultivars ‘Dire’ and ‘Meshkat’ on 6% polyacrylamide mini-protecan procase gel (Bio-Rad, USA). **e** RT-PCR detection of *Arabis mosaic virus* by amplification of the viral 427 bp genome in cultivars ‘Arbequina’, ‘Amin’, ‘Meshkat’, ‘Conservolia’, ‘Tokhm-e-Kabki’, ‘Dire’, ‘KH15’, ‘Shenge’, negative (2) and positive (3) control; **f** Partial amplification of a 513 bp fragment belonging to *Cucumber mosaic virus* genome in cultivars ‘Mastoidis’, ‘Zard’, ‘Meshkat’, ‘Amygdalolia’, ‘KH15’, ‘Coratina’, ‘Dire’, negative (2) and positive (3) controls. Lanes (5) in figure **a** and 2 in **b**, **c**, **e**, **f** indicate negative controls, and lanes 3 in **e** and **f** show positive controls. DNA ladders are 1 Kb plus ladder (Fermentas, USA) for **a**, **b** and **c**; Invitrogen 10 bp DNA ladder for **d** and 100 bp ladder for **e** and **f**

The quantity and quality of RNA samples extracted from olive cultivars using different RNA extraction methods were checked by running small amount of RNA (1 µL) on 1.5% agarose gel. Ethidium bromide staining of agarose gel electrophoresis showed two sharp bands corresponding to 18 S and 28 S rRNA for samples in fresh extractions (Fig. 1a) except for the RNAs extracted by modified CTAB 2 method, which had no sharp bands. In Fig. 1 the prepared cDNA library, partial amplification of housekeeping genes (*Rbc 1* and *Nad 5*), and of *Arabis mosaic virus* (ArMV), and *Cucumber mosaic virus* (CMV) genomes from several cultivars are also shown. All the RNAs extracted manually or by commercial kits showed acceptable quality on gel electrophoresis except for modified CTAB 2 protocol. The housekeeping genes were amplified successfully using related synthesized cDNAs (Fig. 1b, c), except for some repetitions of samples (data are not shown). According to NEBNext^®^ Ultra™ II multiplex small RNA Library Prep set manufacturer’s instruction, the microRNA (miRNA) and Piwi-interacting RNA (piRNA) bands normally range between 140 and 150 bp, respectively while adaptors are ligated (Fig. 1d). The results of RT-PCR amplification of viral genomes from air-dried samples extracted by TRIzol 1 had the most qualified bands (Fig. 1e, f) and sequence (data are not shown).

### The effects of sample procedure, extraction method and cultivar on RNA integrity and quality

**Fig. 2:**
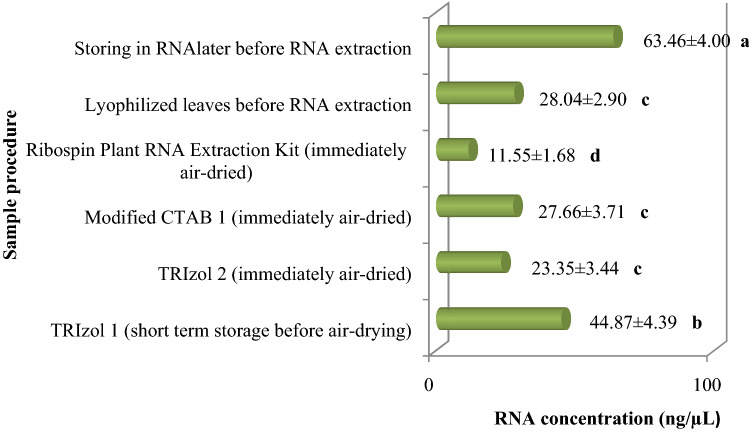
RNA concentrations (ng/µL) of olive cultivars samples transferred out-of-ice (Mean  ±  SE)

Total RNAs extracted from fresh or lyophilized tissues, or recovered from air-dried RNAs shipped in RNAlater (averaged of all cultivars) showed different concentrations (Table 2). TRIzol 1 extracted fresh RNA had the highest RNA concentration mean amongst cultivars (Table 2) which were not able to be transferred without keeping at – 20 °C. Amongst the transmissible RNAs, RNAlater had the highest concentration. The second high concentration belonged to short term storage before air-drying in RNA extracted by TRIzol (TRIzol 1) and resulted in significance differences (Tables 2, 3, Fig. 2). However, the TRIzol 1 method had the best RNA quality in gel electrophoresis and virus genome amplification (Fig. 1), achieved the second highest concentration amongst out-of-ice shipment methods. The least acceptable yield was recorded by Ribospin Plant RNA Extraction commercial kit when the RNAs were immediately air-dried (Fig. 2).

**Table 2:** The comparison of RNA concentrations mean in various fresh and transportable methods

Sample type	Extraction method	Concentration (ng/µL)	CV	A260/280	A260/230
Fresh extractions	TRIzol	455.26 ± 4.54^a^	0.75	1.72 ± 0.04	0.22 ± 0.02
	Modified CTAB 1	330.74 ± 4.38^b^	0.74	2.03 ± 0.27	0.97 ± 0.13
	Ribospin plant RNA extraction kit	33.12 ± 1.2^c^	0.82	1.40 ± 0.13	0.72 ± 0.19
	Modified CTAB 2	14.93 ± 5.3^c^	0.94	1.39 ± 0.14	0.70 ± 0.17
Air-dried extracted RNA	TRIzol 1	44.87 ± 4.85^c^	0.65	2.02 ± 0.03	1.21 ± 0.13
	TRIzol 2	23.35 ± 3.5^c^	0.97	1.91 ± 0.04	0.64 ± 0.14
	Modified CTAB 1	27.66 ± 3.8^c^	0.89	2.10 ± 0.04	1.11 ± 0.16
	Ribospin plant RNA extraction kit	11.55 ± 1.7^c^	0.96	1.96 ± 0.05	1.50 ± 0.13
Complementary techniques	Lyophilized leaves	28.04 ± 3.7^c^	0.46	1.91 ± 0.04	0.64 ± 0.15
	Storing in RNAlater	63.46 ± 4.0^c^	0.28	2.02 ± 0.03	1.20 ± 0.16

Different lowercase letters denote significant differences among RNA concentrations at P ≤ 0.01 by Duncan’s test

**Table 3:** RNA concentrations comparison of various fresh and lyophilized methods in different cultivars

Method of extraction		Cultivar	Concentration (ng/µL)	CV	A260/280	A260/230
Fresh extractions	TRIzol	Amin	562.85	0.48	1.52	01.15
		Meshkat	711.93	0.77	1.73	0.91
		Arbequina	395.26	0.55	1.79	0.95
		Amygdalolia	597.16	0	1.56	1.07
	Modified CTAB 1	Amin	511.84	0.80	2.03	0.97
		Conservolia	254.56	0.43	2.06	0.74
		Megaron	261.4	0.33	2.04	1.18
		Grossane	287.28	0.17	2.06	1.38
	Modified CTAB 2	Meshkat	42	0.94	1.39	0.70
		Amin	19.81	0.57	1.08	0.41
		Arbequina	11.2	0	1.60	0.24
		Dire	18.7	0.16	1.20	0.51
Air-dried extracted RNA	TRIzol 1	Amin	741.933	0.65	2.05	1.21
		Tokhm-e-Kabki	599.28	0.14	1.99	1.37
		Conservolia	608.14	0.04	1.96	0.99
		X3	25.99	0	2.03	1.97
	TRIzol 2	Amin	210.73	0.97	1.91	0.64
		Tokhm-e-Kabki	36.91	0.85	1.89	0.57
		Cornicabra	216.98	0.88	1.68	0.59
		Dire	80.49	0.64	1.86	0.57
	Modified CTAB 1	Amin	377.56	0.89	2.10	1.11
		Grossane	8.87	0	2.17	0.93
		Meshkat	102.63	0.68	1.99	0.96
		Halkidikis	218.51	0.73	1.40	1.16
	Ribospin plant RNA extraction kit	Koroneiki	11.55	0.96	1.96	1.50
Complementary techniques	Lyophilized leaves	Dire	282.89	0.46	1.91	0.64
		Amin	20.61	0.56	1.88	0.95
		Meshkat	59.29	0.80	1.88	0.25
		Arbequina	137.70	0.64	2.06	0.76
	Storing in RNAlater	Amin	526.23	0.27	2.09	1.61
		Meshkat	681.46	0.32	2.09	1.30
		Tokhm-e-Kabki	494.26	0.16	2.03	1.61
		Dire	497.88	0.16	2.05	1.53

For fresh methods they were measured immediately after extraction, in case of transportable methods they were measured after 10 days of air-drying, and in case of complementary methods they were measured after storing at room temperature for 10 days and later were used for RNA extraction (Mean  ±  SE).

A260/280 ratio values of RNA ranging from 2.03 ± 0.27 to 2.10 ± 0.04 in fresh and air-dried RNAs obtained from CTAB 1, which can be considered as the least contaminated in this study (Table 2). Regarding polysaccharide contamination, A260/230 ratios were 0.97 ± 0.13 and 1.11 ± 0.16 in fresh and air-dried CTAB 1 samples, respectively showing not much difference with TRIzol 1 (Table 2). In Table 3, RNA concentrations comparison of various fresh and lyophilized methods in some cultivars are shown. RNA concentrations in case of shipping out-of-ice are also shown in Fig. 2.

### RNA integrity and quality assessments according to Agilent and bioinformatics analysis (Fast QC)

**Fig. 3:**
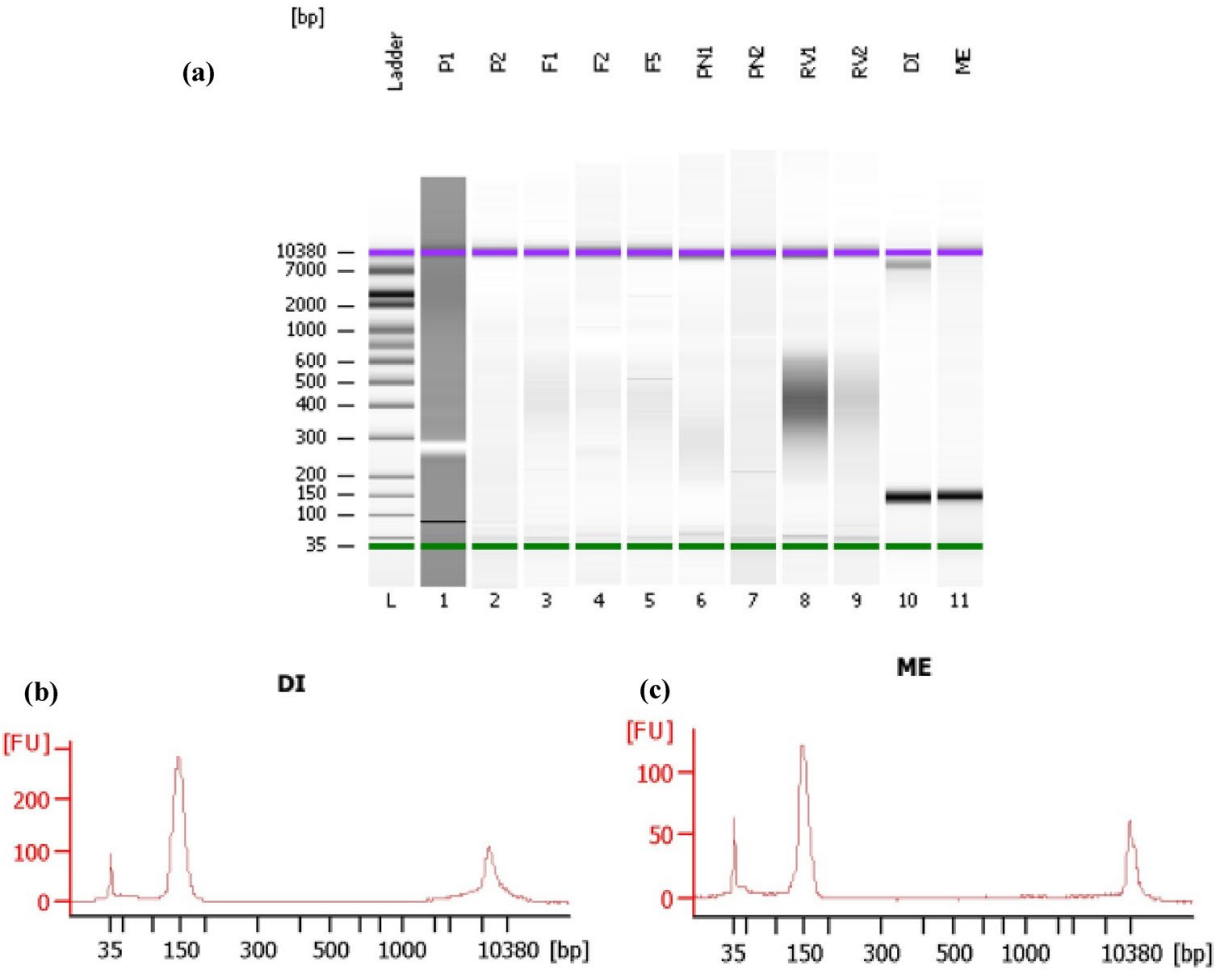
**a** Electrophoresis file run summary and **b** Electropherograms obtained from running the chip by Agilent 2100 (Bioanalyzer, DEU) of ‘Dire’ (DI) and ‘Meshkat’ (ME) samples optimal for high throughput sequencing

The presence of around 150 bp bands is shown in Fig. 2a which were set according to the viruses in another study. In addition, the 5 S, 18 S, 28 S and fast region, pre region, and post region areas can be seen in Fig. 3b, c.

**Fig. 4:**
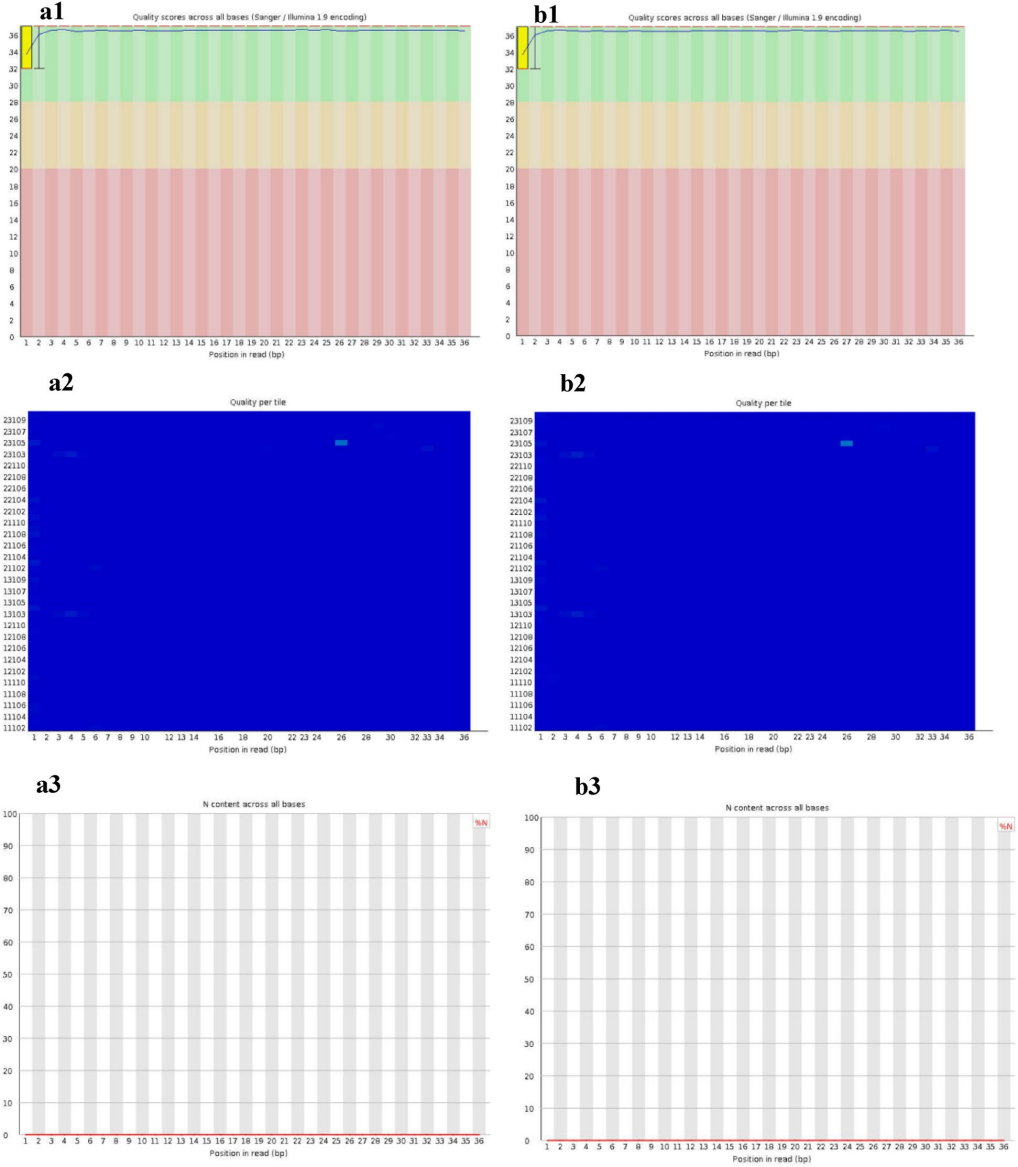
Quality score across all bases, quality per tile and N content across all bases in **a** ‘Meshkat’ (6,329,648 raw reads) and **b** ‘Dire’ (7,002,412 raw reads) sequencing FastQC results

The FastQC results of sequenced metavirome of air-dried RNA samples extracted by TRIzol 1 showed that quality score, known as Phred or Q score, across all bases is an integer value representing the estimated probability of an error showing the base is incorrect. Phred score of 20 or above is acceptable; however in the present study Q score was around 36 except for three starter bases which normally were trimmed during bioinformatics analysis. In this case, an incorrect base call was less than 1 in 1000 meaning more than 99.9% confidence (Fig. 4, a1 and b1). Regarding the quality per tile factor, the colors are on a cold to hot scale, with cold colors being positions where the quality was at or above the average for that base in the run, and hot colors indicate that a tile had worse qualities than other tiles for that base (Fig. 4, a2 and b2). N content across all bases shows that the percentage of bases at each position or bin with no base call, i.e., ‘N’ is zero (Fig. 4, a3 and b3). This module raises a warning if any position shows an N content of  > 5% (Michigan State University Site, https://rtsf.natsci.msu.edu/genomics/tech-notes/fastqc-tutorial-and-faq/).

## Discussion

### The effect of plant tissue on RNA integrity and quality

The cambium scrapings samples showed low RNA yield and higher contamination compared with leaf tissue samples. In contrast to this study, it was claimed that RNAs isolated from leaf tissue produced a faint band in RT-PCR detection of viruses [22]. Nevertheless, these results unequivocally indicate that both leaf tissue and cambium scrapings can serve as reliable sources for the detection of several important grapevine viruses [22], as both parenchymal and vascular viruses are able to be detected in host. Xiao et al. [22] also reported that further grinding of plant tissues in extraction buffer after the initial grinding in liquid nitrogen greatly increased the RNA yield for viral diagnosis targets. Therefore, in the present study a mixture of petioles, leaves and bark tissues were used for higher RNA yield and virus detection accuracy purposes.

### Gel electrophoresis of RNA and PCR products

The height of the 28 S and 18 S rRNA bands could be compared to each other, with a 2:1 ratio indicating non- or slight-degraded RNA. Since 28 S rRNA is normally degraded more quickly than 18 S rRNA, so that a sharp 28 S rRNA band could prove the extracted RNA yield. It is already reported that degradation time of RNAs varies according to the RNA type [23].

### The effects of sample procedure, extraction method and cultivar on RNA integrity and quality

RNA isolation from plant tissues is generally more challenging than animal tissues. The rRNAs and tRNAs are two types of RNA numerously found in all cells and are mostly extracted in the same proportion [24]. It is reported that RNA yield and integrity did not decline in lyophilized human cells stored up to 2 months [3, 25], and acceptable yield and quality of RNA were obtained from dormant grapevine buds (*Vitis vinifera* L. cv. ‘Flame Seedless’) which were lyophilized and stored at room temperature for 0, 3 and 6 weeks [2]. Several stable RNA lyophilisates including sucrose, glucose and mannitol for long-term storage up to 36 months at 25 °C have been applied in medical researches [1]. Lyoprotectants are excipients which prevent catalytic and hydrolytic activities by replacing the hydration sphere around a molecule [1].

However, lipidoid nanoparticles (LNPs) were able to be lyophilized without diminishing their potency [26], further concerns existed about the potency of lyophilized composition obtained by the used method [1]. It is reported that RIN values measured by Agilent in case of using trehalose was the same as non-lyophilized sample [3]. In another study, small interfering RNA-loaded lipidoid-polymer hybrid nanoparticles were spray-dried and the results showed that physicochemical characteristics as well as in vitro siRNA release profile and gene silencing were comparable to non-spray-dried LPNs [27].

In the present study, TRIzol 1 can be considered a suitable method for overseas exchanges and sequencing as it is reported to be efficient specifically in small RNA studies [22]. Noteworthy, RNA concentration is highly dependent on cultivar and the existence of secondary metabolites co-purified in RNA precipitation process. In this study, the method of precipitation also had the biggest impact (Tables 2, 3).

CTAB as a well-known methodology is applicable for a wide range of tissues and species such as apple, pear, potato, thyme, wheat, rice, etc. The accuracy of its results for PCR, RT-PCR, real time-PCR and southern blot analysis have been proved [28]. In this study, CTAB 1 revealed the low contamination with polyphenols, however olive cultivars are highly enriched with phenolic compounds. This effect can be attributed to the presence of PVP 40 in CTAB buffer. The reduction of secondary metabolites due to the formation of large and insoluble complexes with reactive phenolic substances and prevention of complexing with nucleic acids resulting in the increased release of nucleic acids into the homogenate during extraction. Masoomi-Aladizgeh et al. [28] claimed that this protocol can potentially extract both DNA and RNA simultaneously and based on the purpose, a slight alteration will result in either RNA or DNA. Purification can be managed by adding RNase and DNase enzymes or adjustment of the extraction buffer pH. They also recommended this buffer for recalcitrant plants enriched in secondary metabolites.

Abnormal 260/280 ratios usually indicate that a sample is contaminated by residual phenol, protein and guanidine [29]. These types of consideration can lead to small RNA biology studies in woody plant species precipitating from young and old leaves [22]. Similar to this study, it was reported that RNA lyophilisation diminishes the RNA contamination mediated by nuclease and protease activities in samples rich in polyphenol and polysaccharide contents [2]. However, in the present study CTAB 1 revealed more satisfactory results (Table 2). Air-dried RNA of TRIzol 1 method showed acceptable quality and integrity even for sequencing purposes which proves the capability of out-of-ice transferring method. TRIzol extracted RNAs yielding good quality and purity of fresh RNA extraction were also used for virus detection in Greek olive cultivars showing the samples were free of genomic DNA achieved in less than 3 h without the extra need for purification steps [18].

### RNA integrity and quality assessments according to Agilent and bioinformatics analysis (Fast QC)

The RINs greater than 7 are generally considered excellent for use in RNA-Seq application [3]. The results of this study roughly estimated the RINs of 7–9 for the samples, and quality control by Agilent technology further proved the TRIzol 1 method efficiency for metavirome studies in olive cultivars (Fig. 3). Even though the RNAs extracted by other methods namely TRIzol 2, CTAB 1, TRIzol l and commercial RNA extraction kits that were not sequenced, their relative efficiencies for RT-PCR studies (68%, 68%, 61% and 52% of samples, respectively) could estimates their RIN values above 5 that needs to be documented. It is reported that TRIzol kit produced higher yield than the other kits (Qiagen, Bioneer and Sigma) based on the intensity of low molecular weight RNA bands (small RNA) of grapevine (*V. vinifera* cv. Chardonnay) on the gel electrophoresis [22]. Read mappability and library complexity of samples were also highly affected by RNA modifications and high quality and mismatch rates decreased the mapping quality [25].

## Conclusion

Since the application of virus-free planting materials is of great importance in sustainable agriculture, the need to accurately detect plant materials infected with viruses has an applicable significance. Olive genotypes as valuable horticultural products host several viruses from different genera. Molecular biology studies regarding olive total RNA and also phytopathological investigation necessitate RNA stabilization for a longer period.

RNA integrity and value is highly cultivar dependent. For fresh extraction studies, TRIzol reagent performance in olive cultivars RNA mean value was prominent. In shipment cases, despite RNA stabilizer superiority in RNA value, air-dried cases extracted with TRIzol reagent were more satisfactory in agarose gel electrophoresis, cDNA synthesis, library preparationn, and RT-PCR amplification of both housekeeping and virus genes. Although secondary metabolites vary significantly amongst olive cultivars, RNA extraction methods are prominently determinative in contamination co-purification with targeted RNA. CTAB 1 showed an acceptable range of contamination (A260/280 and A260/230), which is highly attributed to its chemistry. Consequently, RNA air-drying can be considered a promising method in the out-of-ice shipment process of plant materials, where there is a scope for improvement.

## Methods

### Plant materials and general procedures

**Fig. 5:**
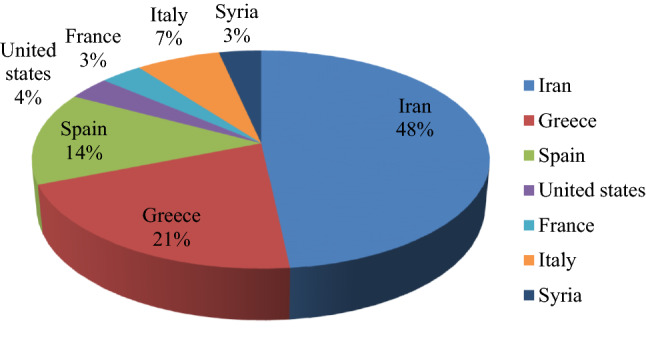
Distribution of olive cultivars from different countries in the present study

Samples of leaves, petioles and bark tissues with three replications were collected randomly from actively growing shoots of 70 mature olive trees with ascertained genetic trueness from Olive Research Station (Tarom, Zanjan province, Iran) in June 2019, possessing the main sources for olive improvement in Iran [30]. We state that we had the permission to collect olive materials from Olive Research Station in order to produce virus free plant materials. The samples consisted of cultivars from Iran [‘Amin’, ‘Meshkat’, ‘Tokhm-e-Kabki’, ‘Dire’, ‘Mary’, ‘Zard’, ‘Shiraz’, ‘BN6’, ‘KH15’, ‘Shenge’, ‘Roghani’, ‘Kolahfaraj’, ‘X3’ (‘Avan’), ‘Ozine2’], Greece (‘Conservolia’, ‘Megaron’, ‘Halkidikis’, ‘Mastoidis’, ‘Amygdalolia’, ‘Koroneiki’), Spain (‘Arbequina’, ‘Cornicabra’, ‘Picual’, ‘Manzanilla’), United States (‘Mission’), France (‘Grossane’), Italy (‘Leccino’, ‘Coratina’) and Syria (‘Abo Satal’) (Fig. 5). All tissues of samples were mixed individually, homogenized to fine powder in liquid nitrogen using an automated tissue homogenizer and cell lyser (Geno Grinder 2010, UK) and kept at − 80 °C. For pathological detection experiments, all stages of sampling, grinding and storage were performed in accordance with the principles of quarantine without interfering with each other.

### RNA extraction methods

All glassware and tips used for RNA extraction were autoclaved twice for 45 min (121 °C, 1.37 bar) unless otherwise stated, and all pliers were oven dried at 200 °C overnight. All chemicals were of analytical grade. To optimize RNA concentration in different tissues, leaf RNA and stem bark of ‘Conservolia’ cultivar were compared and a combination of leaf, petiole and bark tissues were selected for all other cultivars.

#### TRIzol 1 and 2

Total RNA from each sample was extracted using TRIzol™ Reagent (Thermo Fisher Scientific, USA). One millilitre of TRIzol™ was added to 100 mg of homogenized sample, mixed well and centrifuged. The supernatant was mixed with 200 µL chloroform (Merck, USA) and recentrifuged. The resultant supernatant was mixed with 250 µL isopropanol (Merck, USA), and 250 µL NaCl (2 M) (Duchefa Biochemie, NLD) and centrifuged again. Pellet was washed twice with 75% (v/v) ethanol (AR, China), resuspended in 25 µL DEPC (Invitrogen, USA) treated deionized water, incubated at 65 °C for 15 min and stored at − 80 °C. All centrifugations were done for 15 min at 13,000 rpm and 4 °C. In TRIzol 1, RNAs were stored at − 80 °C for a short time before air-drying, whereas in TRIzol 2 samples were immediately air-dried.

#### Modified CTAB 1

This protocol was done according to Doyle and Doyle [31] with some modification reported by Masoomi-Aladizgeh et al. [28]. Briefly, CTAB buffer was prepared by dissolving 0.5 g cetyltrimethylammonium bromide (CTAB) (Biochemica, UK), 1 g EDTA disodium dihydrate (Duchefa Biochemie, NLD), 2.5 g Tris base (Sigma, USA) and 5 g NaCl (Duchefa Biochemie, NLD) in 100 mL autoclaved double distilled water on shaker at room temperature. Next, 15 mg/mL polyvinylpyrrolidone (PVP) 40 (Sigma-Aldrich, USA) and 10 µL/mL β-mercaptoethanol 98% (Sigma, USA) were added to the solution and the CTAB buffer was incubated (60 °C for 20 min). For RNA extraction, 1 mL CTAB buffer and 20 µL β-mercaptoethanol were added to 200 mg fine ground sample, mixed well and kept in bain-marie for 10 min at 60 °C. Then, 600 µL chloroform:Isoamyl alcohol (IAA) (Merck, USA) (24:1) was added to each sample and centrifuged (10 min, 10,000 rpm, 4 °C). Subsequently, 700 µL isopropanol (− 20 °C) (Merck, USA) was added to the supernatant and centrifuged again. Pellet was washed with 1 mL ice cold 96% (v/v) ethanol (− 20 °C) and centrifuged for 2 min at 7500 rpm and 4 °C then was dried in sterile air under chemical hood. Finally, the pellet was resuspended in 45 µL DEPC treated deionized water.

#### Modified CTAB 2

This method was done according to Zarei et al. [29] using two extraction buffers. Extraction buffer I contained CTAB (2% w/v), EDTA (25 mM), Tris–HCl (100 mM, pH 8.0 ± 0.1) (Merck, DEU), NaCl (2 M), spermidine (0.5 g/L) (Sigma-Aldrich, DEU) and PVP 40 (2% w/v). In addition, proteinase K (50 µg/mL) (Qiagene, NLD) and β- mercaptoethanol (20 µL) were added to the buffer after autoclaving for 20 min. Extraction buffer II contained NaCl (1 M), Tris–HCl (10 mM, pH 8.0 ± 0.1), EDTA (1 mM, pH 8.0 ± 0.1) and sodium dodecyl sulfate (SDS) 0.5% (w/v) (Merck, DEU). This buffer was also autoclaved. Both buffers were heated to 65 °C before use.

For RNA extraction, 1 mL pre-heated buffer I was added to 100 mg of fine ground plant tissues of cultivars ‘Amin’, ‘Meshkat’, ‘Tokhm-e-Kabki’, ‘Dire’, ‘Arbequina’, ‘Conservolia’, ‘Koroneiki’ and followed by adding 20 µL β-mercaptoethanol. Tubes were thoroughly vortexed and incubated (15 min, 65 °C) and centrifuged (10 min, 9000 rpm, 4 °C). The supernatant was transferred to new tube and equal volume of chloroform:IAA (24:1) was added, vortexed and centrifuged (10 min, 10,000 rpm) and this step was repeated. Next, ¼ volume of aqueous upper phase, LiCl (1 M) was added to the resultant supernatant and was gently inverted and incubated at 4 °C overnight. The sample centrifuged for 40 min at 12,000 rpm and 4 °C. This supernatant was decanted and tubes were gently tapped on autoclaved tissue paper (Whatman 2, UK). The pellet was washed with 500 µL ice cold 70% ethanol and centrifuged shortly. In the next step, 300 µL pre-heated buffer II was added to dissolve the pellet. An equal volume of 24:1 chloroform:IAA was added and centrifuged for 10 min at 10,000 rpm and 4 °C. Then, 0.1 volume of sodium acetate (NaOAc) (Merck, DEU) (3 M, pH 5.2 ± 0.1) and 0.6 volume of isopropanol were added, mixed and incubated at − 20 °C for at least 2 h. Tubes were centrifuged for 20 min at 12,000 rpm and 4 °C and the pellets were washed with ice cold 70% ethanol and dried for 15 min on tissue paper. The pellets were resuspended in 30 µL PEPC treated deionized water.

#### Commercial RNA extraction kits

Two commercial RNA extraction kits namely Ribospin Plant RNA Extraction Kit (GeneAll, South Korea) and Spectrum Plant Total RNA Kit (Sigma-Aldrich, DEU) were used, according to respected manufacturer’s illustrations.

### Air-drying of precipitated RNA

The extracted RNAs of all cultivars were first adjusted to the same quantity and quality (A260/A280). The RNAs extracted with TRIzol 1, 2, CTAB 1 methods and Ribospin Plant RNA extraction kit, were precipitated with 0.1 volume sodium acetate (3 M) and 5 volume ice cold 100% ethanol. Samples were vortexed thoroughly and kept at − 20 °C overnight. Then, the samples were centrifuged for 30 min at 13,000 rpm and 4 °C. The RNA pellets were washed with 0.5 mL ice cold 75% (v/v) ethanol, span for 10 min and stored at room temperature for 10 days. Air-dried total RNAs were shipped out–of-ice and diluted in respected buffer from Spectrum ™ plant total RNA kit (Sigma-Aldrich, DEU) after 10 days for further analyses.

### Lyophilisation of olive tissues

Samples of leaves, petioles and bark tissues of each cultivar (‘Amin’, ‘Meshkat’, ‘Tokhm-e-Kabki’, ‘Dire’, ‘Arbequina’, ‘Conservolia’) were first ground in liquid nitrogen to fine powder and then lyophilized for 18 h at − 60 °C by vacuum freeze dryer (OPERON, KR). Lyophilized samples were stored at room temperature for 10 days before RNA extraction. RNAs were extracted by Spectrum ™ plant total RNA kit (Sigma-Aldrich, DEU) according to the manufacturer’s instruction.

### Shipment in RNAlater solution

Samples (‘Amin’, ‘Meshkat’, ‘Tokhm-e-Kabki’, ‘Dire’, ‘Arbequina’, ‘Conservolia’) consisting of leaves, petioles and bark tissues were immersed in RNA stabilizer solution (Yekta Tajhiz Azma, IRN) and stored at room temperature for 10 days before RNA extraction by Spectrum plant total RNA method (Sigma-Aldrich, DEU).

### Studying the RNA quality and quantity

The quality and integrity of extracted RNAs were assessed by several methods including spectrophotometry for A260/280 and A260/230, appearance on a denaturing 1.5% agarose gel, ability to amplify RT-PCR products and fluorimetry (Turner BioSystems Modulus Fluorometer, USA) just after elution in both fresh extracted and lyophilized samples (before storage at − 80 °C). The RNA Integrity Number (RIN) were tested by Agilent 2100 (Bioanalyzer, DEU) referring to the amount of different RNAs. An algorithm is generated by taking multiple samples and manually assigning them all a value of 1–10 based on their integrity which 10 represent the least degradation. RQS vs RIN (with 10% CV) is calculated by

$${\text{RQS}} = {\text{A}} + \left( {1 - \frac{Fast Region Area}{{Total Area}}} \right) \times X1 + \left( {\frac{18 S Area + 28 S Area}{{Total Area}}} \right) \times X2 + \left( {\frac{28 S Height}{{18 S Height}}} \right) \times X3$$

where A, X1, X2, and X3 are constants (Caliper Life Sciences, 2009, www.caliperLS.com, Application Note 402).

### Molecular assays for detection of viral and host targets

RNA of all cultivars was subjected to cDNA synthesis using random hexamer primer and M-MuLV reverse transcriptase (Promega, USA) according to manufacturer’s instruction for fresh and transferred samples. The cDNA was tested for several housekeeping genes including *Rubisco* (*ribulose bisphosphate carboxylase chloroplast ribosomal*) with primer pair F-TACTTGAACGCTACTGCAG and R-CTGCATGCATTGCACGGTG [32] and *Nad 5* (mitochondrial gene of higher plants encoding subunit 5 of the NADH ubiquinone oxidoreductase complex), and two olive viruses including *Arabis mosaic virus* (ArMV), and *Cucumber mosaic virus* (CMV) using the previously reported primer paires [33, 34] as qualitative measurement methods. To confirm virus infection, the amplified PCR products were sequenced using 310 data collection software version 3.1.0 on a ABI PRISM TM 310 Genetic Analyzer (Applied BioSystem, USA). They were subsequently cleaned up by NucleoSpin gel and PCR clean-up (Takara Bio, USA), then asymmetric PCR was done by reverse primers and magnetic beads protocol carried out according to BigDye^®^ Sequencing clean-up kit (MCLAB, USA).

The cDNA libraries were prepared using NEBNext^®^ Ultra™ II multiplex small RNA Library Prep set (New England Biolabs, USA) according to the manufacturer’s instructions for MiniSeq Illumina^®^ (New England Biolabs, Ipswich, MA, USA). Regarding Agilent assessment, gel-dye mix was allowed to equilibrate to room temperature for 30 min before use. Next, a new High Sensitivivity DNA chip was put on the chip priming station. Then 9 µL of gel-dye mix was pipetted in the well marked as G. The plunger was positioned at 1 mL and then the chip priming station was closed. Plunger was pressed until it was held by the clip. It was waited for exactly 60 s then clip was released. Next it was waited for 5 s, then the plunger was slowly pulled back to the 1 mL position. The chip priming station was opened and 9 µL of gel-dye mix was pipetted in the wells marked G. Next, 5 µL of marker (green) was pipetted in all sample and ladder wells. One microliter of High Sensitivity DNA ladder (yellow) was pipetted in the well marked as ‘ladder’. Then, 1 µL of sample and marker was pipetted to used wells and unused wells, respectively. Finally, the chip was put horizontally in the adapter, vortexed for 1 min at 2400 rpm, and was run in the Agilent 2100 Bioanalyzer instrument within 5 min.

### Experimental design and statistical analysis

The RNA extraction experiments were conducted under a completely randomized design with at least 3 replications. Data were evaluated by the analysis of variance (ANOVA) according to the general linear model (GLM) procedure using statistical software SPSS Statistics 22 (IBM, USA) and mean comparisons among treatments were conducted using Duncan test at the *P*  ≤ 0.01.

## Data Availability

All data generated or analyzed during this study are included in this published article and its additional files or the datasets used and/or analyzed during the current study are available from the corresponding author on reasonable request.

## Abbreviations

ArMV: *Arabis mosaic virus*
CTAB: Cetyl trimethylammonium bromide
CMV: *Cucumber mosaic virus*
DNA: Deoxyribonucleic acid
DEPC: Diethyl pyrocarbonate
IAA: Isoamyl alcohol
PVP: Polyvinylpyrrolidone
RNA: Ribonucleic acid
SDS: Sodium dodecyl sulfate
